# Potential Distribution of *Pilocarpus microphyllus* in the Amazonia/Cerrado Biomes under Near-Future Climate Change Scenarios

**DOI:** 10.3390/plants12112106

**Published:** 2023-05-25

**Authors:** Waléria P. Monteiro, Everaldo B. de Souza, Leonardo de Sousa Miranda, Luciano J. S. Anjos, Cecilio F. Caldeira

**Affiliations:** 1 Instituto de Geociências, PPG-Ciências Ambientais, Universidade Federal do Pará, Belém 66077-830, PA, Brazil; waleriapmonteiro@gmail.com (W.P.M.); ljsanjos@gmail.com (L.J.S.A.); 2 Lancaster Environment Centre, Faculty of Science and Technology, Lancaster University, Lancaster LA1 4YQ, UK; mirabala@gmail.com; 3 Campus de Parauapebas, Universidade Federal Rural da Amazônia, Parauapebas 68515-000, PA, Brazil; 4 Instituto Tecnológico Vale, Belém 66055-090, PA, Brazil; cecilio.caldeira@itv.org

**Keywords:** climate change impacts, species distribution modeling, Brazilian tropical biomes, CMIP6 scenarios

## Abstract

*Pilocarpus microphyllus* Stapf. ex Wardlew. (Rutaceae) is an endemic and threatened medicinal plant species from tropical Brazil. Popularly known as “jaborandi”, it is the unique natural source of pilocarpine, an alkaloid used to medical treat glaucoma and xerostomia. Based on Species Distribution Models (SDMs), we modeled the suitability of *P. microphyllus’s* geographical distribution considering three Global Circulation Models (GCMs) under two future climate change scenarios (SSP2-4.5 and SSP5-8.5). The quantitative analyses carried out using ten different SDM algorithms revealed that precipitation seasonality (Bio15) and precipitation of the driest month (Bio14) were the most important bioclimatic variables. The results evidenced four main key areas of continuous occurrence of the plant spreading diagonally over tropical Brazilian biomes (Amazon, Cerrado and Caatinga). The near-future (2020 to 2040) ensemble projections considering all GCMs and scenarios have indicated negative impacts for the potential loss or significant reduction in suitable habitats for *P. microphyllus* in the transition region between the Amazon and Cerrado into central and northern Maranhão state, and mainly in the Caatinga biome over the northern Piaui state. On the other hand, positive impacts of the expansion of the plant habitat suitability are projected over forest cover protected areas of the Amazon biome in the southeastern Pará state. Since the jaborandi is of socioeconomic importance for many families in the north/northeast Brazil, it is urgent to implement public policies for conservation and sustainable management, thus mitigating the impacts of global climate change.

## 1. Introduction

Global climate change has turned into one of the most important rising threats to biodiversity on Earth, and there is scientific consensus that plants are among the most vulnerable and threatened groups [1,2,3]. Plants may move, adapt, or go extinct, in the latter case because they are not able to cope as quickly with the increase in surface air temperature and water stress caused by climate change [4,5]. In the face of global changes, the consequent alterations ranging from large to regional scales into environment conditions may directly affect plants’ physiology, phenology, and distribution throughout their diverse ranges [6,7]. Analyzing the impacts of future global changes, particularly on Amazon biodiversity, Gomes et al. [8] projected a reduction between 30% and 47% of all plant species and geographical distribution, with the eastern Amazon basin being the most heavily impacted.

Previous studies have already demonstrated the effect of climate change on medicinal plants. For example, Rana et al. [9] and Shen et al. [10] showed the influence of precipitation, temperature, and elevation on the potential distribution, quality, and habitat suitability of economically important medicinal and aromatic plants in China and Nepal. In addition, medicinal plant species are particularly threatened by climate change, as future conditions can increase the anthropogenic pressure of disordered exploration, leading to the loss of the area of the natural occurrence range [11,12,13].

*Pilocarpus microphyllus* Stapf ex Wardlew. is a medicinal shrub tree, popularly known as “jaborandi” (in Portuguese), recognized as the unique natural source of pilocarpine, an imidazole alkaloid extensively used in the industry as an active pharmaceutical ingredient (API) in the composition of medicaments applied to treat diseases such as glaucoma and xerostomia [14,15,16]. The species plays a significant economic role in generating income for several families that depend on the extraction of non-timber forest products [17,18]. In 2021, the extraction of jaborandi leaves reached approximately 291 tons in Brazil [19], and pilocarpine export generated a revenue of USD 3.3 million in the first half of 2019 [20]. Thus, because of the socioeconomic pressures, *P. microphyllus* is under threat of extinction and was placed in the “Vulnerable” category [21] as a result of disorderly extractive practices. During the last few decades (especially in the 1970s and 1980s), increasing pressure from the overexploitation of *P. microphyllus* has contributed to reductions in the population’s size/abundance throughout its distribution area and its complete disappearance in some regions [15,18,22].

**Figure 1:**
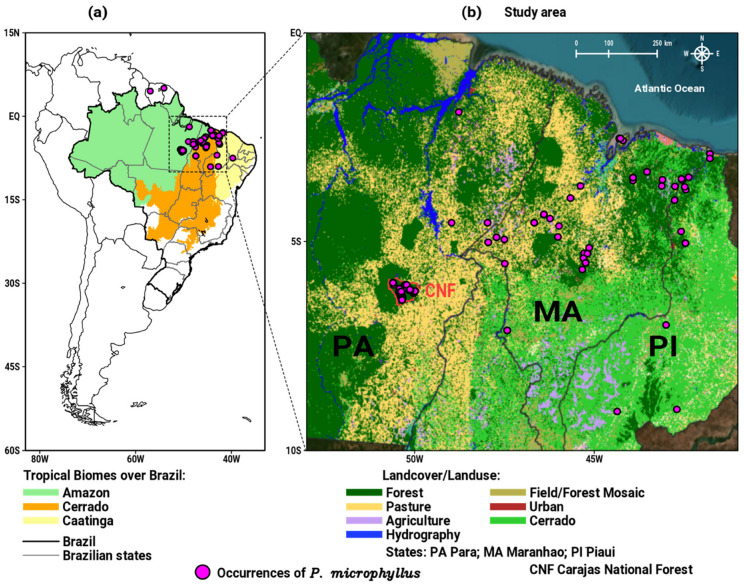
Maps with the occurrence points of *P. microphyllus* (circles) over: (**a**) South America, emphasizing the geographic domain of the three tropical biomes over Brazil; (**b**) study area of the present work in north/northeast Brazil with the landcover/landuse map in the year 2020.

On the South American continent, *P. microphyllus* is native to north and northeast Brazil and to some areas in French Guiana and Suriname (see circles in Figure 1a) [23,24,25]. In the Brazilian territory, this plant has a predominant occurrence in the states of Pará (PA), Maranhão (MA), and Piauí (PI) [26], encompassing the tropical portion of the Amazon, Cerrado, and Caatinga biomes, as shown in Figure 1a. Therefore, the focus of this research is on the study area illustrated in Figure 1b, where the points of occurrence of the *P. microphyllus* are more abundant. MA is the largest producer of jaborandi, followed by PA and PI [19]. PA is considered as one of the largest natural reserves of *P. microphyllus* in the eastern Amazon, where it grows in the restricted area of Carajás National Forest (CNF; see location in Figure 1b), a conservation unit in this state [15,27]. Indeed, a notable environmental feature when viewing the landcover and landuse map in Figure 1b is the presence of extensive areas of pasture (yellow areas) between the Amazon and Cerrado biomes (PA and MA states). The deforestation level has intensified in these areas due the multiple anthropic activities (agriculture, cattle, settlements, and mining), which results in the loss and fragmentation of the plants habitat [28]. The impact of human activities on endangered species is even more serious, as they are more sensitive to climate change, because their habitat is directly influenced by regional climate patterns represented by surface air temperature and precipitation regimes [9,29,30]. The distribution of species around the world depends on a set of abiotic and biotic aspects, including the vegetation types where the species grows; however, in Brazilian tropical biomes, climatic conditions play a relevant role, such that climate change can directly affect the availability of suitable habitats in the future [8,31,32,33].

Few studies have examined how climatic conditions can affect the growth, reproduction, and potential distribution of *P*. *microphyllus*. Lima et al. [34] showed the highest levels of *pilocarpine* in the dry season and lowest levels in the rainy season. More recently, Caldeira et al. [15] found possible suitable areas for the occurrence of P. *microphyllus* but did not evaluate the consequences of climate change on the geographic range of suitable habitats in the future. It is therefore critical to understand how the geographical distribution may respond to climate change, since these species are used by local communities and tend to have high economic value, with high demands on the pharmaceutical industry [18,20].

Species Distribution Modeling (SDM) is a robust scientific and computational technique with a high level of reliability in environmental studies [35,36,37]. SDM has been considered an essential tool to verify the effect of climate changes on the distribution of natural organisms over time [38]. This method uses species occurrence data together with environmental and ecological data, projecting and mapping the places most likely to be suitable for the species. Map projections under future scenarios allow the assessment of the effectiveness of current Protected Areas [39] and may also help scientists and decision makers with the planning of integrated conservation/restoration strategies of biodiversity [40,41].

Here, we apply the SDM approach to assess the suitable habitat areas for *P. microphyllus* under current and different future climate change scenarios. The SDM will help in understanding how climate change affects geographic distribution, with the hypothesis that as air temperature rises and the rainfall regimes are modified in the coming future conditions, some areas might become less or more suitable for *P. microphyllus*. Therefore, the objectives of the study are (i) to determinate the potential distribution of suitable habitats for *P. microphyllus* associated with environmental variables during the current climate; (ii) to investigate the impact of global climate change scenarios on the distribution of occurrence of this species during the next two decades (2020 to 2040).

The state of the art in environmental modeling developed here can contribute to advancing scientific knowledge about this plant of relevance in terms of the biology of conservation as well as for human practices that depend socioeconomically on them. The results should provide us with useful ideas and knowledge for identifying priority regions for the conservation of the species and information to delineate sustainable exploitation areas of the jaborandi, together with the implementation of ex situ conservation programs to protect the genetic diversity of populations of this highly vulnerable species.

## 2. Results

Initially, we evaluated the consistency and quality of environmental modeling for the studied species. In terms of metrics used, the True Skill Statistics (TSS) and Receive Operating Characteristic (ROC) obtained values of 0.923 and 0.993, respectively (see Table A1 in Appendix A). These values indicate high levels of accuracy and suitable predictive power and performance to model validation. The integrations of the ten SDM algorithms revealed that the current distribution of *P*. *microphyllus* is dependent on bioclimatic variables (see Table A2 in Appendix A), with the precipitation seasonality (Bio15) and the precipitation of the driest month (Bio14) being the most important for species across the study area.

**Figure 2:**
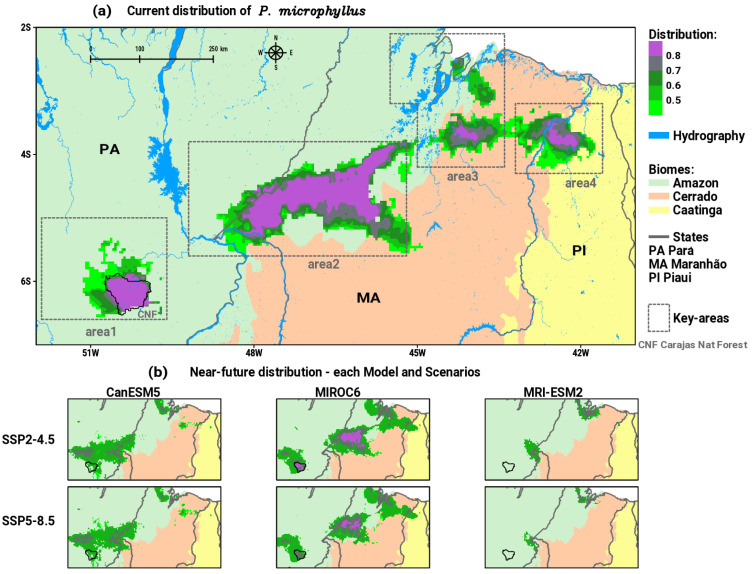
Potential distribution of suitable habitats for *P. microphyllus* (green to purple shaded areas) in the north/northeast Brazil between Amazon, Cerrado, and Caatinga biomes considering: (**a**) current and (**b**) near-future projections for each one of the three GCMs in SSP2-4.5 and SSP5-8.5 scenarios. Polygons in gray dashed lines in (**a**) indicate the key areas of occurrence of the species.

The results obtained in the ensemble model demonstrated that the current distribution of *P*. *microphyllus* presents four main spatially continuous areas (see Figure 2a). Area1 is located entirely in the Amazon biome within the CNF protected area over southeastern PA state. Area2 is the most extensive in the region, along the transition between the Amazon and Cerrado biomes, between the states of PA and MA. Yet, in the transition zone between the Amazon and Cerrado, in the equatorial part of MA, area3 is verified. In the easternmost portion of the region, area4 occurs in the transition zone between the Cerrado and Caatinga biomes, between the states of MA and PI. A certain separation between area1 and area2 can be seen due to the presence of the Tocantins river on the border PA and MA, also between area2 and area3 by the Mearim river in the state of MA.

Concerning the near-future (2020 to 2040) simulations generated using SDM, Figure 2b shows the results obtained for each GCM in the SSP2-4.5 and SSP5-8.5 scenarios, which indicate a general pattern of reductions in suitable areas for the occurrence of *P*. *microphyllus* along the key areas.

**Figure 3:**
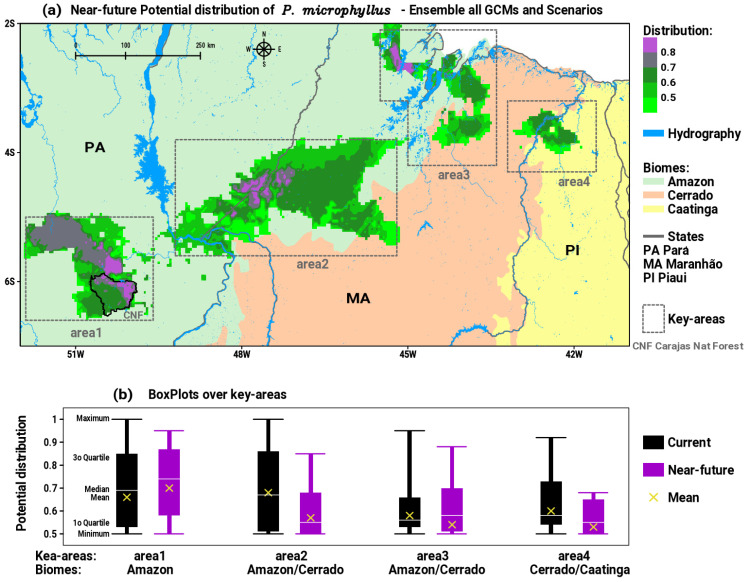
(**a**) Near-future (2020 to 2040) potential distribution of suitable habitats for *P. microphyllus* (green to purple shaded areas) considering the ensemble of all GCMs and scenarios; (**b**) boxplots of the distribution in the four key areas considering the statistics for current and near-future projections.

The analysis of the consensus or ensemble of all GCMs and scenarios for the near future (2020 to 2040) is synthesized in Figure 3a. By comparing the patterns of the current distribution in Figure 2a with the future in Figure 3a, we can distinguish visible changes in area2, area3, and area4 that show decreasing trends both in spatial patterns and in medium to high intensities of suitable habitats for *P*. *microphyllus*. Otherwise, area1 shows an expansion and intensification of suitable areas to the north and northwest, towards protected areas with primary forest cover in southeastern PA. Table 1 exhibits the values of descriptive statistics calculated in each key area of potential distribution of *P. microphyllus*. These results are plotted in Figure 3b as boxplots with the comparison between current and near-future patterns, thus allowing us to extract quantitative information about the impacts of climate change. The effect of environmental changes in the coming decades is to expand area1, maintaining the intensity of the medium to high probability of suitable areas for the species, as indicated by the boxplot for near-future positions relatively above the current distribution (Figure 3b). In area2, which is the most diagonally extensive between the Amazon and Cerrado biomes, there is no significant change in the spatial dimension (Figure 3a); however, the boxplot presents statistics for the near-future period well below the current (Figure 3b), indicating a decrease in the intensity of the plant’s habitats distribution. The simulations for area3 show indications of relative spatial retraction to the south and expansion to the north (Figure 3a), with the position of the near-future boxplot having significant values below the current pattern (Figure 3b). The most intense sign of impact is expected in area4, where a large spatial reduction is clearly configured (Figure 3a) with a consequent robust decrease in the intensity of the suitability of species distribution, as indicated by the boxplot for the current compared to the near-future condition. This result reveals that the suitable habitats for *P. microphyllus* will almost disappear in the Caatinga biome.

## 3. Discussion

The modeling approach using SDM with integrations of various algorithms representing environmental interactions has demonstrated the dominant effect of seasonal precipitation (Bio14 and Bio15) in determining the areas of occurrence of *P*. *microphyllus* along the Brazilian tropical biomes. Four key areas of this species were identified, and near-future projections using the data provided by three GCMs considering two global emissions scenarios (SSP2-4.5 and SSP5-8.5) have indicated substantial spatial retraction trends in potentially suitable habitats for the survival of *P*. *microphyllus,* particularly over transitions regions between the Amazon/Cerrado/Caatinga biomes (central to northern Maranhão and Piaui states). Such negative impacts were associated with future changes in seasonal rainfall regimes in the eastern Amazon and northeast of Brazil.

Our findings are consistent with previous studies of environmental and climate impacts on the most diverse distributions of plant species. Igawa et al. [42] reported the precipitation of the driest month as a main variable for predicting the suitable areas in the distribution of *Theobroma cacao* L., a species of economic importance for many small farmers in Brazil. Centeno et al. [43] demonstrated the importance of precipitation seasonality as a relevant cofactor for determining the distribution of a shrubby cactus, *Tacinga palmadora* (Britton & Rose) N. P. Taylor & Stuppy, endemic to Brazil, in the Caatinga biome. Tian et al. [44] observed a decrease in the suitable regions for *Zanthoxylum armatum* DC. (Rutaceae) and suggested that the specie is susceptible to climate change. In addition, Fragnière et al. [45] have shown that, under future climate scenarios, the suitable areas for many species can be decreased, which might well indicate increased extinction risk. Additional evidence shows that reducing areas of suitable climatic conditions affects foraging resources available for the pollinators, threatening the sexual reproduction and genetic variability of plants [23,46].

Despite the occurrence of *P*. *microphyllus* in northeast Brazil, where the climate is hotter with prolonged dry seasons, antecedent studies have suggested that it is mainly found in the lowlands of the Brazilian Amazon biome [47,48]. According to Caldeira et al. [15], *P*. *microphyllus* is located in Carajás National Forest (CNF), a sustainable federal conservation unit located in the southeast of the state of Pará, eastern Amazon. This region is characterized by precipitation between 2000 and 2400 mm, while the average annual temperature varies from 23 °C and 26 °C [49], which offers to the specie the maturation of its fruits and flowers, and subsequently, good growth [50]. A recent study on *P*. *microphyllus* showed that the increase in temperature and decrease in precipitation projected in RCP scenarios may affect the jaborandi seedlings’ growth [51]. Furthermore, these authors have emphasized the negative impact on growth in the height, diameter, and leaf area of the seedlings caused by water stress. Another study [50] recorded the effects of precipitation and temperature on phenological patterns of *P*. *microphyllus* and showed that fruiting and flowering peaked in the wettest period.

Ribeiro et al. [52] showed significant impacts of climate change on the environmental suitability of *Dipteryx alata* Vogel in the Brazilian Cerrado. Similar results have been reported in other studies of plants species from the Cerrado, related to climate change’s impacts [52,53,54]. However, despite these studies demonstrating negative effects on the distribution of plants in this biome, Tomaz et al. [55] indicated that a medicinal plant species tends to adapt in different environmental conditions, since the distribution of the species tends to decrease in the Atlantic Forest and Caatinga over northeast Brazil.

So, there is scientific evidence that global to regional changes in the environmental conditions may negatively impact medicinal plants, causing a decrease in suitable habitats, changes in productivity of plants, and even promoting the most serious case of species extinction [11,56]. Considering the importance of these plants to *pharmaceutical industry* [20], it is expected that climate change will negatively impact the regional economy. For example, in the state of Pará in the eastern Amazon, collecting *P*. *microphyllus* leaves has become an important income source for several families and has driven the local economy [28,57]. According to the IBGE [19], in the state of Pará, the extraction of jaborandi leaves moved approximately 38t/year, referring to plant extraction carried out in Brazil in the year 2021. From a socio-economic point of view, the income produced by the extractivism of jaborandi currently generates around BRL 2500 (monthly income/member); however, the family tend to participate during the harvest period, which may possibly contribute to an increase in family income [58]. Our results point to an expansion of suitable habitats for this species in southeastern Pará, mainly around the CNF. The projections for the southeastern Amazon, including the CNF area, indicate an intensification of the rainy season, especially in the SSP2-4.5 scenario. So, this region will not be impacted by a reduction in the plant, but sustainable management plans are recommended as they are within protected areas with primary forest cover. Amaral et al. [51] demonstrated the impact of climate change on jaborandi seedlings’ growth, thus directly affecting their future production. Our results corroborate these authors, but the most significant negative impacts should occur in the transition regions between the Amazon and Cerrado biomes along the state of Maranhão and primarily on the border with the state of Piaui already within the Caatinga biome, whose projections of spatial retraction and intense decreases in suitable habitats of the plant could really compromise the economy of many families in these two states of north and northeast Brazil.

## 4. Materials and Methods

### 4.1. Studied Species, Study Area, and Collection Data

*Pilocarpus microphyllus* Stapf ex Wardleworth is a shrub or small tree that belongs to the Rutaceae family. The species is perennial diploid (2n = 22), allogamous and reaching a height of 3–6 m [24,48]. *P*. *microphyllus* are found in the South American continent (Figure 1a), particularly in north and northeast Brazil, more precisely, over the states of Pará, Maranhão, and Piauí (Figure 1b) [26,59,60]. *P*. *microphyllus* grows in regions with low to high altitudes (between 50 and 550 m) and milder air temperatures and a well-defined rainy season [61]. Figure 1b shows that the study area encompasses the occurrence of *P*. *microphyllus* along the tropical portion of the Amazon and Cerrado biomes (Pará and Maranhão), as well as a small area of the Caatinga biome (western Piaui) [62,63]. So, the Amazon and Cerrado biomes are the most significant areas of *P*. *microphyllus* occurrences in Brazil. Each biome presents distinct climatic characteristics; for example, in the Amazon, the climate is hot and humid, with high rainfall and air humidity throughout the year [63,64]. Cerrado has dry winters and rainy summers, while Caatinga presents semi-arid conditions with a short rainy season [62,65].

The occurrence records of *P*. *microphyllus* used in this study were extracted from the *SpeciesLink* database (https://specieslink.net, accessed in 15 January 2022), as well all similar data described by Monteiro et al. [66]. First, the data from the *SpeciesLink* were checked to verify whether all the occurrence points really belonged to *P*. *microphyllus*, and whether there were duplicate and/or missing data. In addition, the geographic coordinates were verified, since there may have been inconsistencies related to possible typing errors. After this step, the occurrence points located in the states of Maranhão and Piauí were taken from the *SpeciesLink* database, while the points over the state of Pará were taken from Monteiro et al. [66]. It is important to emphasize that we only used records of the species that occur naturally in the study area (Figure 1b) [26,59,60].

### 4.2. Bioclimate Variables in the Current Climate and Future Scenarios

For this study, the 19 bioclimatic parameters were selected for modeling in the current climate scenario, obtained from the WorldClim database version 2.1 [67]; (http://www.worldclim.org, accessed on 28 January 2022), with a 30 arc-seconds resolution (~1 km). First, the “removeCollinearity” function of the virtualspecies package in R [68]; [R Development Core Team, 2019] were used to eliminate the bioclimatic variables that were correlated (r > 0.85) with each other. Among the 19 variables tested, 10 remained in the diagnostic analyses, including: mean diurnal range (Bio2), isothermality (Bio3), temperature annual range (Bio7), precipitation of coldest quarter (Bio9), mean temperature of warmest quarter (Bio10), precipitation of driest month (Bio14), precipitation seasonality (Bio15), precipitation of wettest quarter (Bio16), precipitation of warmest quarter (Bio18), and precipitation of coldest quarter (Bio19).

Concerning the impact analysis of future climate change on the distribution of *P*. *microphyllus* over a 20-year period (2021–2040), we used simulations of Global Circulation Models (GCMs) from the Coupled Model Intercomparison Project Phase 6 (CMIP6; 33, 88). Three GCMs were used: CanESM5, MRI-ESM2-0, and MIROC6, which reproduce the spatial pattern of precipitation for South America best, according to Rivera and Arnould [69] and Almazrou et al. [70]. In terms of future climate conditions, two *Shared Socio-Economic Pathway* (SSP) scenarios were used: SSP2-4.5, which represents moderate projections, as it suggests an increase in global air temperature between 0.9 and 2 °C (considering global mitigation policies to reduce greenhouse gas emissions); and SSP5–8.5, which represents the pessimistic scenario, which suggests an exponential increase in CO_2_ emissions throughout the 21st century and a global temperature increase between 1.4 and 2.6 °C. In this research, GCM data for future scenarios projections (2021–2040) were also obtained from the WorldClim database.

### 4.3. Species Distribution Modelling (SDM)

In the modeling approach, the occurrence data of *P*. *microphyllus* were used as the response variable and bioclimatic and environmental data as predictor variables into the “Biomod2” package developed in the R environment [71]. The last version Biomod2 (3.5.1) was used to identify the more probable areas of occurrence of species through the intersections and interactions between biophysical and environmental factors [72]. To reduce uncertainties produced by biases and limitations of the individual use of statistical techniques, we applied new module routines of Biomod2 that use multiple modeling techniques and allow for the generation of ensemble predictions based on consensuses between models [36]. Thus, for model adjustments, we employed up to ten different modeling algorithms: four machine learning algorithms (generalized boosting modeling—GBM [73]; artificial neural network—ANN [74]; classification tree analysis—CTA [75]; Random Forest—RF [76]) and six regression algorithms (generalized linear modeling—GLM [77]; generalized additive models—GAM [78]; Bioclim—BC [79]; flexible discriminate analysis—FDA [80]; multivariate adaptive regression splines—MARS [81]; maximum entropy—Maxent [82]).

For this study, since there are records of the presence of the plant and for most statistical models it is necessary to have data on the presence and absence of the species, pseudo-absence points sampled throughout the study area were generated. In terms of reducing modeling uncertainties, we adopted the calculation of a consensus procedure (*ensemble*), so the results could be consistent with different modeling studies [83]. An evaluation of the model performance was carried out by using the TSS (True Skill Statistics) metric and the ROC (Receive Operating Characteristic) curve. Since TSS is the most applied metric as a simple, more robust, and intuitive measure of the performance of species distribution models, we used this metric to evaluate the best models to compose the consensus, so that only models with TSS ≥ 0.6 were considered to compose the ensemble [84]. Thus, the consensus distribution model was obtained through the statistics between the best models. Furthermore, we binarized the current and future distribution maps using the maximized threshold values of TSS to transform them into maps of absence/presence ranging from 0 to 1 [85]; however, our results will be emphasized for values above 0.5 containing the highest probability of suitable areas for the studied species. The results were presented separately for each one of the three GCMs in the SSP2-4.5 and SS5-8.5 scenarios, and the ensemble map considering the integration of all GCMs and scenarios was plotted and analyzed. The latter, we consider as the main result of this study, as it contemplates relevant bioclimatic and environmental variables in the determination of continuous areas containing the highest probability of occurrence of suitable habitats for *P*. *microphyllus* along the tropical biomes in north/northeast Brazil. The mapping seeks to identify the key areas suitable for the occurrence of the species. In terms of the quantitative assessment, descriptive statistics were calculated in each key area followed by plotting the results in boxplot form, thus allowing comparisons of current and future patterns. All analyses and results were conducted in computational and scientific tools based on the R environment (version 4.2.2, Team R Core, 2022), and QGIS v. 3.22.4 (QGIS Development Team, 2021).

## 5. Conclusions

The SDM-based environmental modeling approach was successful in determining four main key areas containing suitable habitats for the current distribution of *P. microphyllus* across the Brazilian tropical biomes of Amazonia, Cerrado, and Caatinga.

Based on the results of a robust ensemble projection constructed with bioclimatic variables representing the consensus seasonal precipitation from three GCMs (CanESM5, MRI-ESM2-0, and MIROC6) under two global climate change scenarios (SSP2-4.5 and SSP5-8.5), we conclude that the most severe impacts for the potential loss of or significant reduction in suitable habitats for *P. microphyllus* will be in the transition region between the Amazon and Cerrado biomes into central and northern Maranhão, and also mainly in the Caatinga biome in northern Piaui, where the probable almost complete disappearance of this plant is expected in the next two decades (2020 to 2040). Conversely, positive impacts of the future expansion and intensification of the habitat suitability for *P. microphyllus* are projected for areas of the Amazon biome in southeastern Pará, where primary forest vegetation cover still prevails within the mosaic of protected areas (Carajás National Forest and others).

The aforementioned information and knowledge about the current and future distribution of *P. microphyllus* should be considered by decision makers to mitigate the impacts of climate change. The recommendation to create new protected areas (primary forest areas help directly in the expansion of plant species) should be an environmental priority, particularly in the state of Maranhão. Since the jaborandi plant is of socioeconomic importance for many families in north/northeast Brazil (related to the extractive exploitation of non-timber products), it is urgent to implement public policies for conservation and sustainable management, as suggested worldwide, from researchers in different continents, for other threatened plant species [86,87,88], together with the creation of ex situ conservation programs to protect the genetic diversity (germosplam bank) of populations of this endangered species.

## Figures and Tables

**Table 1:** Statistics calculated in each key area of potential distribution of *P. microphyllus*.

Key-Areas	Period	Minimum	1st Quartile	Median	Mean	3rd Quartile	Maximum
area1	Current	0.50	0.53	0.69	0.66	0.85	1.00
	Near-future	0.50	0.58	0.74	0.70	0.87	0.95
area2	Current	0.50	0.51	0.67	0.68	0.86	1.00
	Near-future	0.50	0.51	0.55	0.57	0.68	0.85
area3	Current	0.50	0.53	0.56	0.58	0.66	0.95
	Near-future	0.50	0.51	0.58	0.54	0.70	0.88
area4	Current	0.50	0.54	0.58	0.60	0.73	0.92
	Near-future	0.50	0.50	0.55	0.53	0.65	0.68

## Data Availability

All databases (sources and references) were described in the Materials and Methods section.

## Appendix A

**Table A1:** Analysis of modeling performance using the TSS metric and ROC curve.

Statistics	Cutoff	Sensitivity	Specificity	Calibration
TSS	452.0	94.370	97.451	0.918 *
ROC	452.3	94.372	97.450	0.993 *

* Significant value.

**Table A2:** Bioclimatic variables and statistics of the current distribution.

Bioclimatic Variables	Statistics
Precipitation of coldest quarter (Bio9)	0.0821
Mean temperature of warmest quarter (Bio10)	0.177
Precipitation of driest month (Bio14)	0.211 ^1^
Precipitation seasonality (Bio15)	0.286 ^1^
Precipitation of wettest quarter (Bio16)	0.0799
Precipitation of warmest quarter (Bio18)	0.0917
Precipitation of coldest quarter (Bio19)	0.0523
Mean diurnal range (Bio2)	0.161
Isothermality (Bio3)	0.158
Temperature seasonality (Bio4)	0.0975

^1^ Significant value.
